# Resting State EEG Directed Functional Connectivity Unveils Changes in Motor Network Organization in Subacute Stroke Patients After Rehabilitation

**DOI:** 10.3389/fphys.2022.862207

**Published:** 2022-04-05

**Authors:** Ileana Pirovano, Alfonso Mastropietro, Yuri Antonacci, Chiara Barà, Eleonora Guanziroli, Franco Molteni, Luca Faes, Giovanna Rizzo

**Affiliations:** ^1^ Istituto di Tecnologie Biomediche, Consiglio Nazionale delle Ricerche, Segrate, Italy; ^2^ Dipartimento di Ingegneria, Università di Palermo, Palermo, Italy; ^3^ Centro Riabilitativo Villa Beretta, Ospedale Valduce, Costa Masnaga, Italy

**Keywords:** EEG, brain sources, causal connectivity, partial direct coherence, directed coherence, stroke, subacute, motor network

## Abstract

Brain plasticity and functional reorganization are mechanisms behind functional motor recovery of patients after an ischemic stroke. The study of resting-state motor network functional connectivity by means of EEG proved to be useful in investigating changes occurring in the information flow and find correlation with motor function recovery. In the literature, most studies applying EEG to post-stroke patients investigated the undirected functional connectivity of interacting brain regions. Quite recently, works started to investigate the directionality of the connections and many approaches or features have been proposed, each of them being more suitable to describe different aspects, e.g., direct or indirect information flow between network nodes, the coupling strength or its characteristic oscillation frequency. Each work chose one specific measure, despite in literature there is not an agreed consensus, and the selection of the most appropriate measure is still an open issue. In an attempt to shed light on this methodological aspect, we propose here to combine the information of direct and indirect coupling provided by two frequency-domain measures based on Granger’s causality, i.e., the directed coherence (DC) and the generalized partial directed coherence (gPDC), to investigate the longitudinal changes of resting-state directed connectivity associated with sensorimotor rhythms α and β, occurring in 18 sub-acute ischemic stroke patients who followed a rehabilitation treatment. Our results showed a relevant role of the information flow through the pre-motor regions in the reorganization of the motor network after the rehabilitation in the sub-acute stage. In particular, DC highlighted an increase in intra-hemispheric coupling strength between pre-motor and primary motor areas, especially in ipsi-lesional hemisphere in both α and β frequency bands, whereas gPDC was more sensitive in the detection of those connection whose variation was mostly represented within the population. A decreased causal flow from contra-lesional premotor cortex towards supplementary motor area was detected in both α and β frequency bands and a significant reinforced inter-hemispheric connection from ipsi to contra-lesional pre-motor cortex was observed in β frequency. Interestingly, the connection from contra towards ipsilesional pre-motor area correlated with upper limb motor recovery in α band. The usage of two different measures of directed connectivity allowed a better comprehension of those coupling changes between brain motor regions, either direct or mediated, which mostly were influenced by the rehabilitation, revealing a particular involvement of the pre-motor areas in the cerebral functional reorganization.

## 1 Introduction

Stroke is the second-leading cause of death and a major cause disability worldwide (Feigin, 2021) and, in particular, ischemic stroke constituted 62.4% of all new strokes in 2019. Focal stroke lesions affecting either cortical and sub-cortical neurons or descending fiber tracts may affect the neural processing in the motor network (Ward and Cohen, 2004; Stinear et al., 2007). This leads to motor deficit, mainly caused by hemiparesis, that is one of the most common stroke-related disabilities (Katan and Luft, 2018). Nevertheless, it has been observed that patients with initially mild to moderate motor impairments may show substantial recovery of the motor function within the first 3 months after stroke onset. This may be driven by neural plasticity and reorganization, which can occur in the brain also far from the lesion site (Nudo, 2006; Cramer, 2008; Rehme and Grefkes, 2013).

To investigate cortical functional reorganization, connectivity models based on the concept that the brain is organized in specialized anatomically distinct areas functionally integrated in networks (Friston, 2002) were applied to neuroimaging techniques, both in experimental and computational simulation studies (Falcon et al., 2016; Allegra Mascaro et al., 2020). Functional connectivity is usually inferred by means of correlations among measurements of neuronal activity neglecting information on the coupling direction, whereas the directed functional connectivity investigates the causal influence that a brain region exerts over another (Friston, 2011; Barrett and Barnett, 2013). In the literature, the analysis of cortical directed and undirected functional connectivity has been extensively used to characterize the brain connections and investigate rehabilitation effects on patients after stroke events (Desowska and Turner, 2019). Connectivity studies can be carried out during both task and resting state conditions, the latter being the easiest protocol executable also for patients in the acute stage. Interestingly, it has been demonstrated that changes in resting state connectivity, especially in the motor network, are predictive of motor function recovery in stroke patients (James et al., 2009; Grefkes and Fink, 2011; Volz et al., 2016; Hordacre et al., 2020).

Among neuroimaging techniques, functional Magnetic Resonance Imaging (fMRI) was one of the first employed to investigate brain connectivity. Given its high spatial resolution and the provided volumetric information, fMRI allows investigation of connections involving both cortical and deep structures in the brain (Farr and Wegener, 2010). Electroencephalography (EEG) has been proposed as a valid alternative technique, as it allowed to reconstruct known resting state networks from signals acquired by scalp electrodes in healthy subjects (Mantini et al., 2007). Despite its lower spatial resolution, EEG offers a higher time resolution with respect to fMRI, allowing to better track the fast reconfiguration of neuronal networks and to extract information on the spectral characteristics of the functional networks related to specific brain rhythms. Moreover, it exploits cheaper and portable instrumentation, allowing measurements both on healthy and pathological subjects, also accessible in complex medical setting (Borich et al., 2015).

From a methodological point of view, a plethora of measures and indexes have been proposed to infer both functional and causal connectivity from EEG recordings (Pereda et al., 2005), including bivariate and conditional Granger causality (GC), transfer entropy (TE), directed transfer function (DTF), directed coherence (DC), partial directed coherence (PDC) and dynamical Bayesian inference. Each of these metrics may be sensitive to different aspects of the neurophysiological data, evidenced either in time or frequency domains or through linear or non-linear relationships. Therefore, the choice of a metric over another depends on the particular aspect of interest in the connectivity network; moreover, the reliability of a specific metric can depend on different factors related to the specific application, such as the stationarity of the signal, non-linear effects or indirect connections among networks nodes. Different causal connectivity metrics can lead to different results and careful interpretation is required. Usually, only one metric is chosen to retrieve networks’ topology and infer the spectral content of the information flow between network nodes. However, the use of a single metric cannot guarantee a comprehensive description of the network (Blinowska, 2011). For instance, GC and TE provide compact information on the overall information flow between network nodes, while DTF, DC and PDC focus on specific rhythms of physiological interest (Sameshima and Baccalá, 2014; Seth et al., 2015); bivariate GC/TE and DC detect the overall flow of information from one node to another, while conditional GC/TE and DTF/PDC elicit direct connections but may lose ability to capture the coupling strength (Faes et al., 2012). Finally, the dynamical Bayesian inference approach has been proposed to assess the cross-frequency coupling strength both in neural networks and among different physiological systems, e.g., cardio-respiratory-cortical interactions (Stankovski et al., 2016). Until now, an agreed standard measure to assess EEG-based causal connectivity is not univocally employed in the literature, and the selection of the most appropriate measure still represents an open issue.

Moreover, in the context of EEG analysis, causal connectivity was often identified directly from the electrodes signal, but it has been demonstrated that connectivity computed in the electrode space is affected by the scalp volume-conduction effect and the obtained results can be hardly interpreted as real brain connectivity (Hassan and Wendling, 2018; Marinazzo et al., 2019; Van de Steen et al., 2019). On the contrary, meaningful connectivity patterns can be derived from cortical source activities extracted from EEG inverse solutions (Michel and Murray, 2012; Haufe et al., 2013).

Although the effects of stroke on resting-state brain connectivity have been widely investigated, especially in the acute phase with respect to healthy subjects, a limited number of works focused specifically on longitudinal effects of motor rehabilitation on the reorganization of the cortical motor network after stroke. Moreover, the majority of studies exploited fMRI to assess functional, non-directed connectivity (Desowska and Turner, 2019). Considering EEG analysis, up to now, the majority of studies explored functional connectivity in post-stroke populations in both acute (Caliandro et al., 2017; Vecchio et al., 2019a, 2019b; Fanciullacci et al., 2021; Hoshino et al., 2021) and chronic stages (Gerloff et al., 2006; De Vico Fallani et al., 2009, 2017; Wu et al., 2015; Hordacre et al., 2020; Molteni et al., 2020; Waterstone et al., 2020; Romeo et al., 2021); only fewer studies investigated the resting-state causal connectivity after stroke (Guo et al., 2014; Pichiorri et al., 2015, 2018; Calabrò et al., 2018; Nicolo et al., 2018; Lin et al., 2021; Yuan et al., 2021). The few studies cited above employing EEG rarely focused on the investigation of the motor network, preferring a large-scale connectivity analysis of the whole brain with synthetic descriptors. Even though this type of information can serve as a general indication on the state of brain’s connections after stroke, information about power spectral strength or flow direction in the identified connections are often missing. This type of information can be obtained by separate connectivity metrics computed from electrodes or brain sources signals and whose underlying interpretation should be carefully considered to compare the results between different studies.

Considering all these aspects, the investigation of motor network directed functional connectivity by means of EEG in sub-acute stroke patients should be deepened from both methodological and application perspectives. To this aim, in this work we perform an EEG-based longitudinal study of resting-state causal connectivity in a population of sub-acute stroke patients with motor impairment who underwent a rehabilitation treatment. We focus on the characterization of the motor network exploiting the cortical source signals reconstructed from EEG scalp recordings and using two different connectivity metrics, i.e., the Directed Coherence (DC) and the generalized Partial Directed Coherence (gPDC). Combining the information carried by these two measures, we aim to provide a description of the variations induced by treatment in both the topography and the transfer of spectral power along the identified functional connections, investigating also possible correlations with the clinical assessment of motor recovery.

In Section 2, a detailed description of the participants enrolled for this study and the methodology followed is presented. In Section 3, the obtained results are reported and their discussion can be found in Section 4. Section 5 draws conclusions of the work.

## 2 Materials and Methods

### 2.1 Population Description

Eighteen subjects (age 67 ± 10, seven females and 11 males) in the sub-acute stage after ischemic stroke were enrolled for this study. Inclusion criteria were age less than 80 years old, first unilateral stroke, distance from acute event less than 30 days, with no other concomitant orthopedic or rheumatologic disease, without global or comprehension aphasia, right-handed according to the Edinburgh Handedness Questionnaire. Each subject was evaluated twice: firstly, at the admission to the rehabilitation center (T0, on average after 12 ± 5 days from the stroke event) and secondly at the end of the rehabilitation treatment (T1, on average after 55 ± 11 days from the event). In Table 1, details about participants’ characteristics are reported. To participate, each subject signed a written informed consent. The study was reviewed and approved by the local Ethics Committee “Comitato Etico Provinciale dell’Insubria” and was conducted in compliance with the Declaration of Helsinki.

**TABLE 1:** Participants’ characteristics.

Subject’s ID	Age (years)	Gender	Lesion side/location	T0 (days)	T1 (days)	FMA_T0_	FMA _T1_	FAC_T0_	FAC _T1_
1	65	M	L frontal CS	14	42	54	66	1	5
2	61	M	L pons	9	55	0	48	0	2
3	65	F	R internal capsule	6	49	38	60	4	4
4	81	F	R paramedian pons	7	49	4	48	0	2
5	70	M	R centrum semiovale	8	50	45	58	0	3
6	81	M	R lacunar	8	57	44	66	1	5
7	53	M	R median cerebral artery	17	89	0	5	0	0
8	74	F	R cerebellum	20	66	44	52	2	3
9	80	M	L lacunar	11	53	24	56	4	5
10	78	M	R internal capsule	7	51	0	7	0	1
11	72	F	L fronto-parietal	12	61	42	62	0	3
12	76	M	R Sylvian/posterior artery	10	48	6	66	1	4
13	52	M	L fronto-parietal	12	44	47	66	2	4
14	65	F	L Pons	27	72	0	0	0	2
15	56	F	R frontal/basal ganglia	11	53	0	2	0	0
16	63	M	R Pons	7	47	38	53	0	3
17	59	F	L median cerebral artery	10	58	38	49	1	3
18	54	M	R bulbous pyramid	12	49	12	57	0	4

Abbreviations: F, female; M, male; R, right; L, left; CS, cortico-subcortical; FMA, Fugl-Meyer Assessment scale; FAC, Functional Ambulation Category.

Patients followed a rehabilitation treatment for both upper and lower limb recovery: each patient underwent to a rehabilitation treatment twice per day, each session lasting 45 min. Training was tailored according to patients’ residuals motor capacity and it included exercise for upper and lower limbs. Lower and upper limbs stretching, lower and upper limbs muscle strengthening, static and dynamic balance exercises, proprioception exercises, trunk control exercise, gait at the parallel bars or in clinical open spaces were the exercise performed.

### 2.2 Functional Assessment

Clinical evaluations were performed by clinicians, i.e., physiotherapist and medical doctor. To assess the upper limb performance the Fugl-Meyer Assessment (FMA) (Fugl-Meyer et al., 1975) before and after treatment was used. The FMA motor score ranges from 0 (hemiplegia) to 66 (upper limb normal motor performance). As for the lower limb, the level of walking ability was assessed by the Functional Ambulation Category (FAC). This gait assessment scale distinguishes six levels of walking ability based on the amount of physical support required. The lowest level 0 indicates a patient who is not able to walk, while level 5, indicates a patient who can walk everywhere independently, including stairs (Holden et al., 1984). Participants’ scores assigned at T0 and T1 for both clinical scales, i.e., FMA_T0_, FMA_T1_, FAC _T0_ and FAC _T1_, are reported in Table 1.

The variations ΔFMA = FMA_T1_–FMA_T0_ and ΔFAC = FAC_T1_–FAC_T0_ was considered as primary clinical outcomes of subject’s motor recovery.

### 2.3 EEG Data Acquisition

Resting state EEG recordings were performed with the Neuroscan system (Compumedics Neuroscan, Compumedics, NC, United States) using 64 Ag/AgCl electrodes placed according to the International 10/20 standard system. EEG data were acquired at a sampling rate of 1,000 Hz, with the reference electrode placed between Cz and CPz positions and the ground electrode positioned anterior to Fz. During the acquisition, patients were asked to keep their eyes closed in a sitting relaxed position for at least 5 min. As for functional assessment, EEG recordings were repeated twice, before (T0) and after (T1) the rehabilitation treatment.

### 2.4 EEG Data Analysis

**FIGURE 1:**
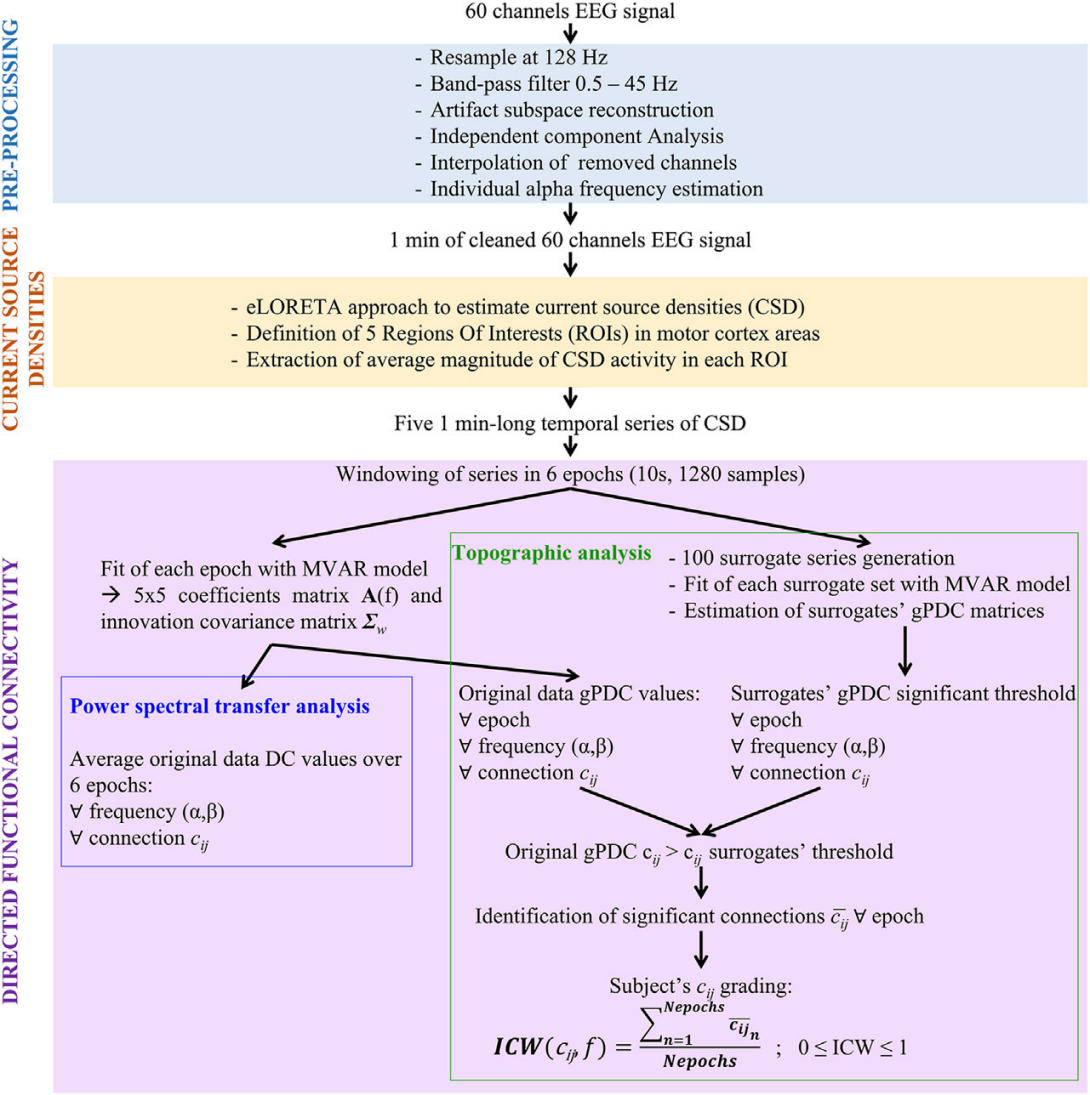
Pipeline of single acquisition and analysis. Abbreviations: MVAR, multivariate auto-regressive; DC, Directed Coherence; gPDC, generalized partial directed coherence.

In Figure 1, a scheme of the principal steps followed for the analysis of single EEG recordings is reported. Three main stages can be identified: EEG signal pre-processing, current sources reconstruction and directed functional connectivity. In the scheme, inputs and outputs of each block are highlighted. Detailed explanation of each step is provided in the following sections.

#### 2.4.1 EEG Pre-Processing

EEG signals were pre-processed offline in Matlab environment (The Mathworks, Inc.) using the open source EEGLab signal processing Toolbox (Delorme and Makeig, 2004).

Data were down-sampled at 128 Hz and band-pass filtered between 0.5 and 45 Hz to remove slow drifts and high-frequency components, such as the power line noise. Flat and “bad” channels were removed for further analysis. A flat channel was identified if it showed amplitude around zero (flatline) for more than 5 s consecutively, whereas a channel was identified as “bad” if it was noisy for more than 90% of the acquisition duration. An Artifacts Subspace Reconstruction (ASR) algorithm (Mullen et al., 2015) was applied to all retained channels to identify and remove transient or high-amplitude artifacts, e.g., eye movements. According to Chang and others (Chang et al., 2020), the application of this cleaning approach improves the accuracy of subsequent Independent Component Analysis (ICA). The ASR algorithm automatically identifies clean periods of the EEG data using a sliding window. An artifact rejection threshold is calculated, based on the calibration signal distribution of variance and a user-defined cut-off parameter. Data portions, whose variance is larger than this threshold, are removed and reconstructed from neighboring channels using a covariance matrix computed from the calibration data. According to the cut-off parameter *k* chosen by the user, the method can be more or less aggressive in removing EEG components portions of data. In this work, *k* = 20 was employed, according to the range suggested by Chang and others, who validated the ASR method on real EEG data and found that a *k* in the range 10–100 allows removing ocular artifacts still preserving cerebral components (Chang et al., 2020). Furthermore, ICA was applied to the ASR-cleaned EEG recordings exploiting the RUNICA Infomax algorithm as implemented in EEGLab (Makeig and Bell, 1996). RUNICA un-mixes the EEG recordings in cortical and non-cortical spatially distinct and temporally independent components (IC). Once the non-cortical components are identified and removed, the final cleaned EEG signal can be reconstructed. To guide the selection of ICs accounting for non-brain (artifactual) activities the ICLabel plugin was employed (Pion-Tonachini et al., 2019). It is an automated classifier which provides an estimation of the type of each of the IC (brain, eye, muscle, line noise, etc.) based on some component characteristics, i.e., power spectrum, its time-course, topography dipole localization and percentage data variance accounted in the considered component. A final visual check of the signals was performed. The originally removed “bad” channels were then reconstructed by the interpolation of the neighbor channels signals, thus obtaining a uniform EEG spatial sampling. Finally, EEG data were re-referenced to a common average reference and 1 min of cleaned, artifact-free 60 channels EEG was selected for all subjects.

#### 2.4.2 Individual Alpha Frequency

To improve the accuracy of the EEG analysis in the frequency domain, the interindividual variability of the alpha rhythm peak was considered for the determination of the spectral bands of interest, as proposed by Klimesch in 1999 (Klimesch, 1999). In particular, for the purpose of this study, we focused our analysis on alpha (α) and beta (β) frequency bands, which are reported as the most involved in motor system functions (Pfurtscheller et al., 1994, 1996). After computing the power spectral density (PSD) for the parieto-occipital channels by the Welch method (Welch, 1967), the Individual Alpha Frequency (IAF) was determined as the average peak position in the spectral range 7.5–12.5 Hz. The average IAF found in our population was equal to 9.07 ± 0.99 Hz. The IAF was used to define the alpha (α) and beta (β) frequency bands as follow: α = [IAF-2 Hz ÷ IAF+2 Hz] and β = [IAF+2.5 Hz ÷ IAF+20 Hz].

#### 2.4.3 Brain Source Reconstruction

Brain source signals were reconstructed from the 60 scalp recordings by means of the exact low resolution brain electromagnetic tomography (eLORETA) method (publicly available free academic software at http://www.uzh.ch/keyinst/loreta.htm). It is a discrete, linear, weighted minimum norm inverse solution, which allows to compute the cortical three-dimensional distribution of current source density (CSD) with exact localization. A detailed description of the method can be found in (Pascual-Marqui, 2007; Pascual-Marqui et al., 2011). eLORETA computations were made in a realistic head model (Fuchs et al., 2002) using the MNI152 template (Mazziotta et al., 2001). The three-dimensional solution space was restricted to the cortical gray matter, as determined by the probabilistic Talairach atlas (Lancaster et al., 2000). The intracerebral volume considered was partitioned in 6,239 voxels at 5 mm spatial resolution. Thus, the neuronal sources electric activity at each gray matter voxel in MNI152 space was reconstructed. In the software, anatomical labels as Brodmann areas (BA) are also available within the MNI space, with coordinates correction to Talairach space (Brett et al., 2002), and were used to identify the Regions Of Interest (ROIs) of this study.

Since in this work we were interested in the study of the resting state motor network, we focused our analysis on five ROIs corresponding to the left and right primary motor cortex (M1), left and right pre-motor cortex (pMC) and supplementary motor area (SMA). To determine the ROIs, all voxels within 8 mm of radius from a given seed were considered as belonging to the specific ROI, thus obtaining ROIs of about 2 cm^3^ volume. The seed coordinates were chosen according to a previous fMRI-based resting state effective connectivity study in stroke patients (Inman et al., 2012). In Table 2, details about ROI centroids coordinates, number of voxels and corresponding BA are provided.

**TABLE 2:** Regions of interest (ROIs) centroids coordinates in MNI coordinates system.

ROI	Position respect to lesion (Acronym)	x	y	z	Broadman area	Number of voxels
L Primary motor Cortex	Contra-lesional (cM1)	−33.0	−19.8	52.1	4	12
R Primary motor Cortex	Ipsi-lesional (iM1)	35.7	−18.1	52.0	4	13
L Pre-motor Cortex	Contra-lesional (cpMC)	−34.3	−1.4	55.8	6	13
R Pre-motor Cortex	Ipsi-lesional (ipMC)	35.1	0.1	54.9	6	13
Supplementary Motor Area	Midline (SMA)	0.0	−4.2	64.7	6	10

The magnitude of the estimated CSD, averaged among all voxels included in a specific ROI, was extracted for each time point, to obtain five time series of brain activity. In order to achieve a homogeneous representation of the population, for each subject the right cerebral hemisphere was considered as the one affected by stroke (ipsi-lesional) and the left hemisphere as the unaffected one (contra-lesional). Hence, for those participants whose brain lesions were in the left hemisphere, the left and right ROIs’ time series were inverted.

#### 2.4.4 Directed Functional Connectivity Analysis

##### 2.4.4.1 Measures Definition

To describe the causal connectivity within the motor network, we calculated two spectral measures of causal flow between two time series: the Directed Coherence (DC) (Baccalá et al., 1998), and the generalized Partial Directed Coherence (gPDC) (Baccala et al., 2007). Both measures are derived from a description of the multivariate CSD series based on linear Multivariate Autoregressive (MVAR) modeling of multiple time series; while the gPDC can be considered as a frequency-domain representation of direct causality between two series in agreement with the concept of Granger causality (Granger, 1969), the DC reflects both direct and indirect causal effects (Faes et al., 2012).

Given a set of *M* time series observed in *N* points, $X\left(n\right)=\;{\left[x_{1}\left(n\right),\;.\;.\;.,\;x_{M}\left(n\right)\right]}^{T},n=1,\ldots ,N,$ the underlying multivariate process can be represented by a MVAR model:

$$X\left(n\right)=\sum_{k=1}^{p}A\left(k\right)X\left(n-k\right)+W\left(n\right)$$

where *p* is the model order, A(k) is an *M*×*M* matrix of coefficients *A*_*ij*_(*k*), describing the dependence of $x_{i}\left(n\right)$ on $x_{j}\left(n-k\right)$ at lag *k* (*i,j* = 1, … ,*M*), and W(n) is an *M* × 1 vector of multivariate uncorrelated white Gaussian noises with *M*×*M* covariance matrix $\Sigma =E\left[w\left(n\right)w{\left(n\right)}^{T}\right]$ .

Applying the Fourier Transform (FT) to the MVAR model leads to its frequency domain representation:

X(f)=A(f)X(f)+W(f),

where X(f) and W(f) are the FTs of X(n) and W(n) , and $A\left(f\right)=\sum_{k=1}^{p}A\left(k\right)e^{-j2\pi fkT}$ provides the spectral representation of the coefficients ( $T=1/f_{s}$ is the sampling period).

Defining the transfer matrix $H\left(f\right)={\left[I-A\left(f\right)\right]}^{-1}=\bar{A}{\left(f\right)}^{-1}$ , the Directed Coherence (DC) is the complex-valued function defined as:

$$DC_{ij}\left(f\right)=\frac{{\text{σ}}_{j}H_{ij}\left(f\right)}{\sqrt{\sum_{m=1}^{M}{\text{σ}}_{m}^{2}\left|H_{im}{\left(f\right)}^{2}\right|}},$$

where ${\text{σ}}_{m}^{2}$ is the *m*th diagonal element of the input covariance Σ ; in the case of strict causality (i.e., diagonal Σ ), the argument under the square root in the equation above is the power spectral density of the *ith* series. The squared modulus of the DC, ${\left|DC_{ij}\left(f\right)\right|}^{2}$ , is a normalized function (ranging from 0 to 1, indicating respectively absence and full presence of causal connectivity) which reflects causality in the frequency domain intended as the portion of the spectrum of the *i*th time series that is causally determined by the *j*th series.

Elaborating the elements of the spectral coefficient matrix in a similar way to that followed above for the transfer function to define the DC, the complex-valued generalized Partial Directed Coherence (gPDC) is defined as:

$$gPDC_{ij}\left(f\right)=\frac{{\text{σ}}_{j}^{-1}{\bar{A}}_{ij}\left(f\right)}{\sqrt{\sum_{m=1}^{M}{\text{σ}}_{m}^{-2}{\left|{\bar{A}}_{mj}\left(f\right)\right|}^{2}}}.$$

The squared modulus of the gPDC, ${\left|gPDC_{ij}\left(f\right)\right|}^{2}$ , is again a normalized function ranging from 0 to 1; the difference with the DC is that gPDC reflects direct causality in the frequency domain, thus eliciting only the direct (i.e., non-mediated) connection from the *j*th to the *i*th series, and in the fact that normalization is performed with respect to the outflow of information from the *j*th series, rather than to the inflow of information towards the *i*th series as happens for the DC.

In this study, the 5 CSD time series of each ROI were epoched in shorter windows of 10 s, thus obtaining six epochs of *N* = 1,280 samples for each subject’s acquisition. The series corresponding to each 10 s-long time frame were fitted with the MVAR model, thus computing the spectral DC and gPDC measures for each acquisition of each subject. Finally, the average DC and gPDC values within the α and β frequency bands as defined in Section 2.4.2 were calculated. Therefore, six values of DC and six values of gPDC for two frequency bands were obtained for each subject in each acquisition (T0 and T1).

As to the selection of the model order *p*, the value yielding the minimum of the Bayesian Information Criterion (Schwarz, 1978) on average for the analyzed population’s series and the number of spectral peaks detectable were considered, leading to the choice of *p* = 8, fixed for all models fitted.

##### 2.4.4.2 Transfer of Spectral Power Within Motor Network Connections Based on DC

As stated in the previous section, the DC values computed for each pairwise connection *c*_*ij*_ provide information about the portion of spectral power that is transmitted from the *j*th to the *i*th time series along all possible patterns of interaction. This measure, averaged inside specific frequency bands, was thus taken as indicative of the “total” effects along each analyzed causal direction. Since no major variation in power content is expected over 1 min of resting state acquisition, for each pair *ij* the DC values was averaged within the α and β bands over the six epochs. This led to perform a statistical comparison between the DC value obtained at the population level for T0 (DC_T0_) and T1 (DC_T1_). A non-parametric one-tailed Wilcoxon’s test for paired samples with significance level equal to 0.05 was used to compare T0 and T1 population’s DC values in both frequency bands. Correction for multiple comparisons (MC) was performed by the False Discovery Rate (FDR) approach (Benjamini and Hochberg, 1995).

##### 2.4.4.3 Topographic Identification of the Motor Network Based on gPDC

In the literature, network analysis has been used in several works to investigate structural, functional undirected or directed brain connectivity at the level of subject populations’ (Rubinov and Sporns, 2010). A network is a mathematical representation of a real-world complex system defined by a collection of nodes (vertices) and links (edges) between pairs of nodes. In this study, the nodes represent the selected ROIs of the motor cortex reported in Table 2, while links are the direct connections identified by gPDC values significantly larger than 0. In practical analysis, measures of causal coupling may present non-zero values which do not reflect existence of real causality between time series but are rather the result of finite size effects on the estimates, or of some feature of the signals itself. Therefore, a statistical surrogate data approach was applied to verify the significance of a connection.

###### 2.4.4.3.1 Surrogate Data Analysis

The method of surrogate data for hypothesis testing (Theiler et al., 1992) consists in testing the considered measure against a null hypothesis, in this case the absence of causal coupling between pairs of time series. To test this hypothesis, a set of surrogate series is generated so that the statistical properties of the original signals are preserved, while being otherwise uncoupled. The considered coupling measure, i.e., here the gPDC, is tested against the null hypothesis through a comparison between its value calculated for the original series and its distribution obtained from the surrogate series. Given a significance level α, a threshold for statistical significance is set at the (1−α) × 100 percentile of the surrogates’ empirical distribution. gPDC values for original data above this threshold can be considered significant and the directed connection $\bar{cij}$ going from the *i*th to the *j*th series is identified as present in the network.

In this work, 100 sets of surrogate time series were generated using the Iterative Amplitude Fourier Transform (IAAFT) algorithm (Schreiber and Schmitz, 1996), which preserves the power spectrum of the original signals while the Fourier phases of the data are randomized, thus destroying the causal coupling. A significance level α = 0.01 was used to identify the threshold of statistical significance from the surrogates’ distribution.

###### 2.4.4.3.2 Single Acquisition Subjects’ Grading

Since we windowed the original 1 min-long time series into six epochs before performing the MVAR model fitting, we obtained six values of gPDC for each subject at both T0 and T1 in both frequency bands α and β. To take into account the possible differences of the synchronization in the EEG signals over the entire minute, we defined an Individual Connection Weight (ICW) as the proportion of number of significant connections $\bar{cij}$ over the six epochs:

$$ICW\left(c_{ij},\bar{f}\right)=\frac{\sum_{n=1}^{Nepochs}{\bar{c_{ij}}}_{n}\left(\bar{f}\right)}{Nepochs},$$

where Nepochs=6 and $\bar{f}$ indicates α or β frequency bands.

**FIGURE 2:**
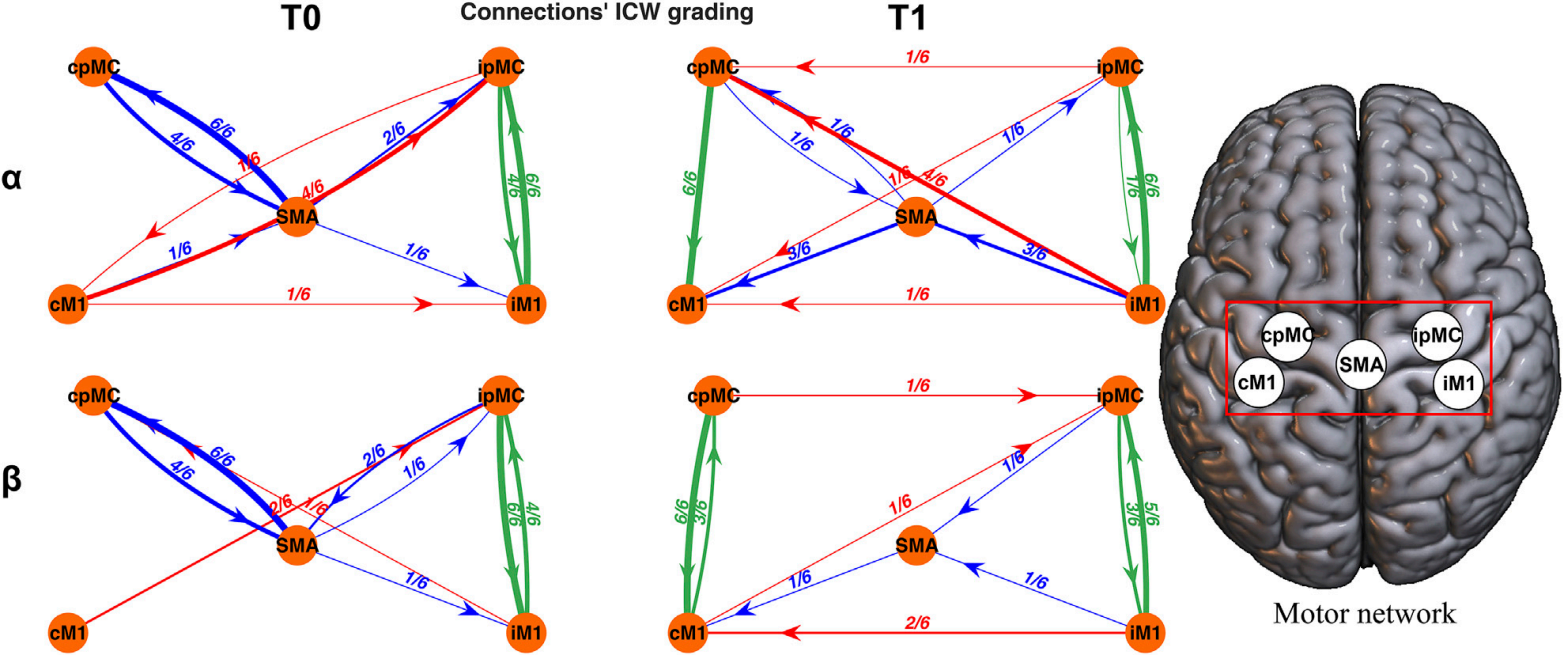
Topographic representation of the motor network reconstructed for a representative subject. Each connection is weighted by ICW. Networks in the α (first row) and β (second row) frequency bands, at T0 (left column) and T1 (right column) are reported. Green arrows: intra-hemispheric connections; red arrows: inter-hemispheric connections; blue arrows: SMA connections.

In this way, *ICW*(*c*_*ij*_) ranges from 0 to 1, with *ICW*(*c*_*ij*_) = 0 if the connection *c*_*ij*_ was not significant in any of the six epochs considered and *ICW*(*c*_*ij*_) = 1 if *c*_*ij*_ was detected as significant in all the six epochs. Thus, the ICW represents a grading of all the subjects’ connections *c*_*ij*_ according to presence of significant causal connectivity between every possible pair of nodes *i* and *j*. In Figure 2, the motor network graph representation for a representative subject is pictured at T0 and T1, for α and β frequency bands, using ICW as weight for the links between nodes. Where the connection is missing, the ICW was equal to 0.

###### 2.4.4.3.3 Assessment of Topological Network Changes and Correlations With the Functional Outcome

To compare the percentage of significant connections in the whole population, i.e., $\frac{ICW\left(c_{ij},\bar{f}\right)}{N_{subjects}}\;\times 100$ , in T1 with respect to T0 condition, the non-parametric McNemar Test for binomial data (Mcnemar, 1947) was employed. As a result, those connections for which the ICW grading significantly varied in the population were detected separately for the α and β frequency bands. The statistical significance level was set at 0.05.

Subsequently, to investigate the possible correlation of the changes in the proportion of significant motor network links before and after rehabilitation with the functional outcome, the Spearman’s correlation between the variation of percentage $\text{ICW}\left(c_{ij},\bar{f}\right)$ , the ΔFMA_T1-T0_ and the ΔFAC_T1-T0_ was computed (significant level = 0.05).

All statistical test results were corrected for MC with FDR technique (Benjamini and Hochberg, 1995).

## 3 Results

In this section, results obtained for the directed functional connectivity analysis of the motor network in our population of sub-acute stroke patients following a rehabilitation treatment are reported. Firstly, results about the directed transfer of spectral power are presented. Topographic results follow in the subsequent subsection.

### 3.1 Motor Network Power Spectral Information

**FIGURE 3:**
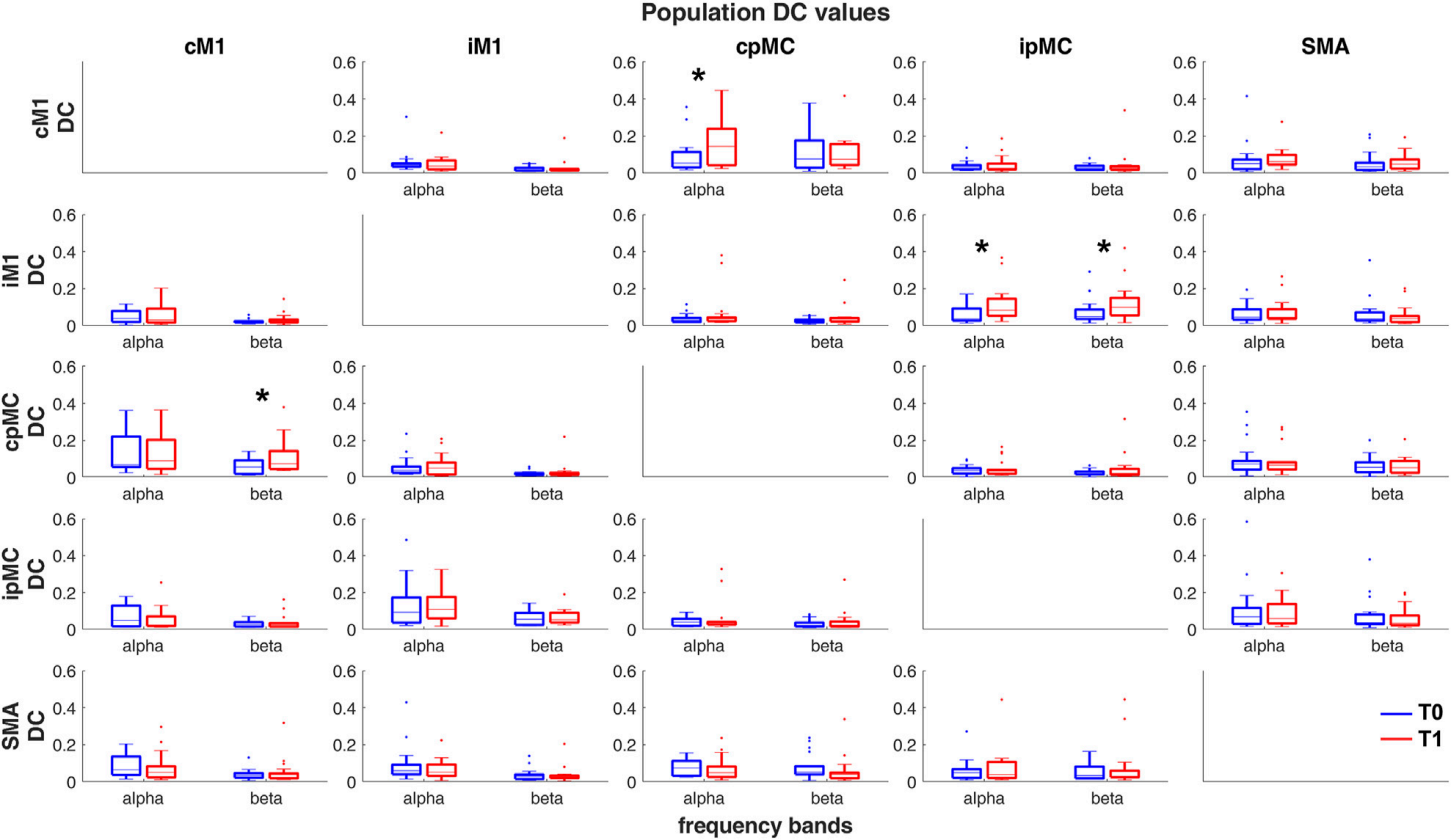
Population’s DC values calculated in α and β frequency bands, in T0 (blue) and T1 (red) acquisitions. Each sub-plot represents a pair of ROIs connection $c_{\text{ij}}$ . Columns *j*: senders; rows *i*: receivers. *Significant variations DC_T1_ - DC_T0_ (one-tailed paired Wilcoxon’s test, *p* < 0.05, without correction for MC).

In Figure 3, population’s DC values calculated for all possible paired connections (subplots) between the 5 motor network ROIs are represented by boxplots. On average, no remarkable differences can be found between DC values for the α and β frequency bands. Focusing on the difference between T1 and T0, a significant increase (Wilcoxon’s test *p*-values < 0.05) in DC_T1_ with respect to DC_T0_ can be observed for the intra-hemispheric connections. In particular, an increase is observed in the α frequency band from pre-motor towards primary motor areas, in both ipsi- and contra-lesional hemispheres (*p*-values = 0.04 and 0.03 respectively). In the β band, the increases in the ipsilesional and contra-lesional sides have opposite direction: ipMC → iM1 (*p*-value = 0.037) and cM1 → cpMC (*p*-value = 0.004). To be noticed, once FDR is applied for correction of MC, none of these variations result as statistically significant.

### 3.2 Motor Network Topography

**FIGURE 4:**
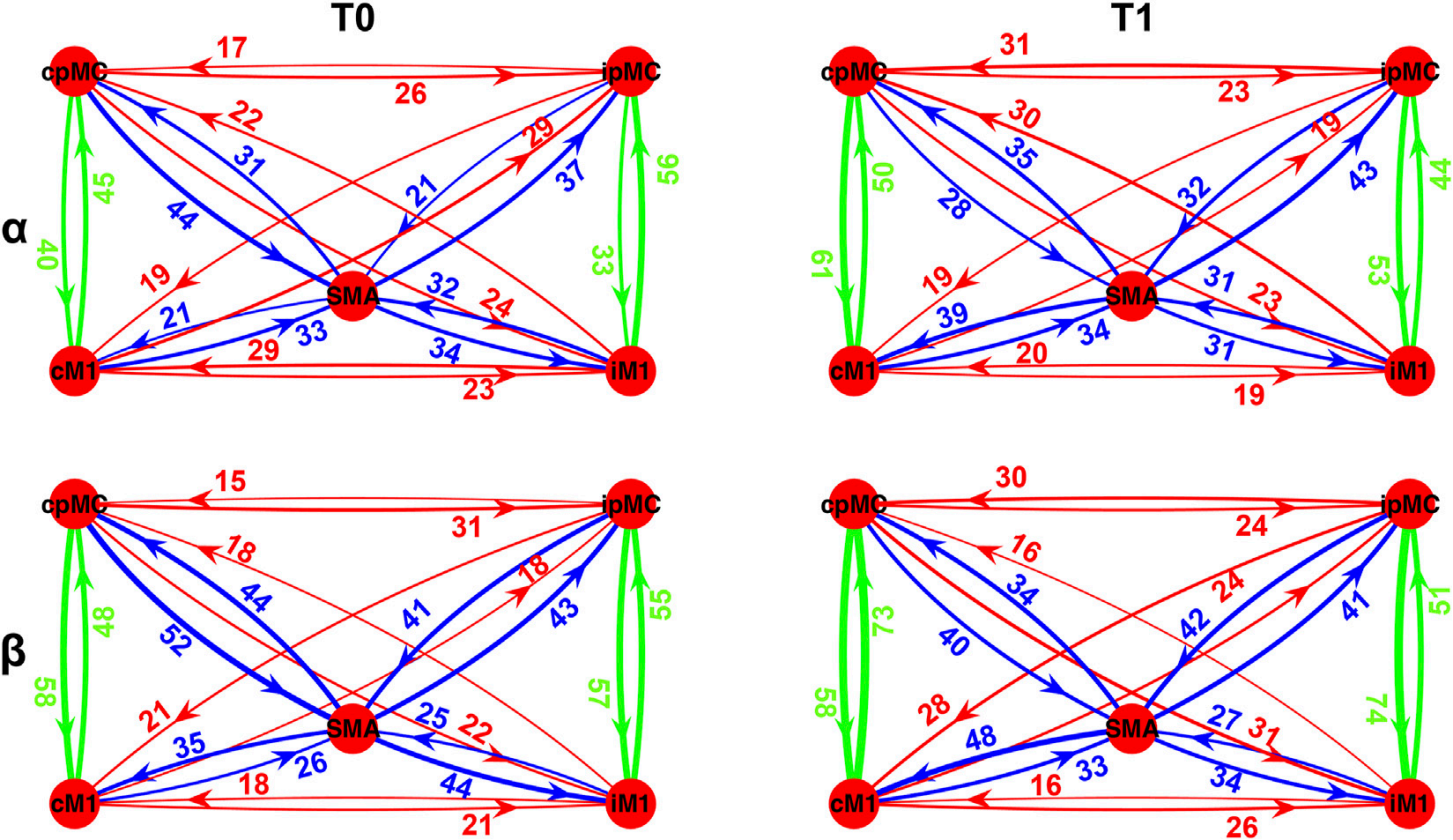
Population’s subject-weighted-topographic connectivity maps in α (top panel) and β (bottom panel) frequency bands, at T0 (left column) and T1 (right column). Weights represent the percentage of subjects for which significant arrow with respect to its surrogate was detected, weighted by ICW. Green arrows: intra-hemispheric connections; red arrows: inter-hemispheric connections; blue arrows: SMA connections.

In Figure 4, a graphical representation of the network topology in T0 and T1 conditions for both analyzed frequency bands is reported. Each connection is weighted by the percentage of its representation in the whole subjects’ population according to the ICW index. It can be noticed that the most represented connections within the population are the intra-hemispheric ones (green arrows), in a percentage ranging from 33 to 61% in the α frequency band and from 48 to 74% in the β frequency. Overall, 20–50% of the subjects showed connection from/to SMA region (blue arrows), whereas the inter-hemispheric ones (red arrows) are less represented, in a range from 15 to 30% of subjects.

**FIGURE 5:**
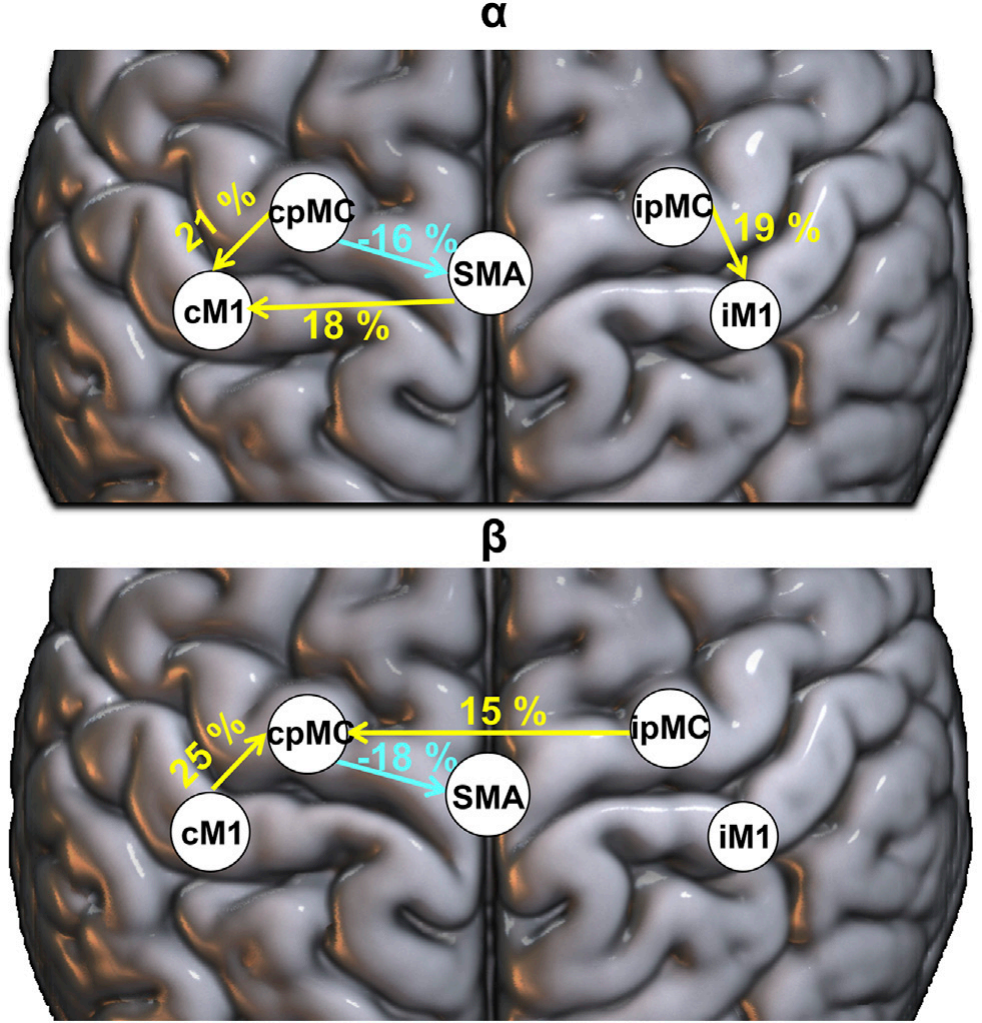
Population’s significant Δ_T1-T0_ ICW connection in α (top panel) and β (bottom panel) frequency bands. Percentages: variation (T1-T0) in the proportion of significant subjects’ connections (weighted for ICW), reported in yellow (greater number of significant connections is present in T1 with respect to T0) and light blue (smaller number of significant connections is present in T1 with respect to T0).

To identify those connections for which the ICW grading changed significantly after the rehabilitation (T1) with respect to the baseline (T0), the McNemar test for proportions significance (*p*-value < 0.05, with MC correction) was performed, and a graphical representation of the results is reported in Figure 5. In particular, we represent only those connections for which, in the whole population, a significant variation emerged in the proportion of epochs where the connection $c_{ij}$ was significantly detected (Δ _T1-T0_% ICW). In the α band, the ICW significantly increased for both ipsi- and contra-lesional intra-hemispheric connections pMC → M1, of 19% (*p*-value = 0.012) and 21% (*p*-value = 0.006) respectively. As for SMA, an increase of 18% in α frequency band in the connection from SMA towards cM1 (*p*-value = 0.001) was found. On the contrary, a decrease of 16% was observed in the number of significant connections from cpMC towards SMA (*p*-value = 0.012). The same connection showed a decrease of 18% also in the β frequency range (*p*-value = 0.015). The major increase in the β band was found only for one of the intra-hemispheric connections (cM1 → cpMC increased of 25% with *p*-value = 0.0001). Additionally, the proportion of inter-hemispheric connections between the two pre-motor areas, from ipsilesional to contra-lesional side, significantly increased of 15% (*p*-value = 0.018).

### 3.3 Correlations to Functional Assessment

**FIGURE 6:**
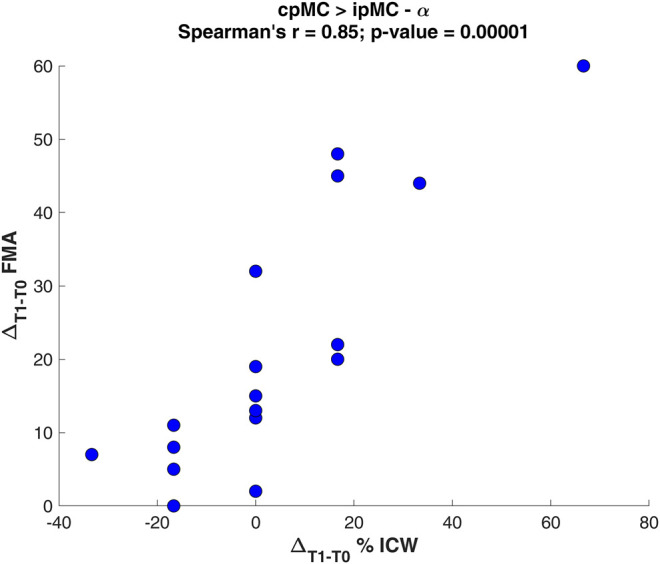
Scatter plot of the Spearman’s correlation analysis between the variation Δ_T1-T0_ of percentage ICW for the connection cpMC→ipMC in α and the Fugl-Meyer Assessment variation ΔFMA_T1-T0_.

From the Spearman’s correlation analysis, we found that the variation of subjects’ ICW grading for the inter-hemispheric connection between the pre-motor areas in α frequency, from contra-lesional to ipsi-lesional hemisphere, positively correlated with ΔFMA_T1-T0_ (Figure 6). Hence, in the examined population, as the proportion of significant connections cpMC → ipMC over 1 min of EEG recording increased after rehabilitation, the motor deficit recovery improved as well.

Conversely, no correlation with the variation of DC magnitude was found. Furthermore, no correlations between functional connectivity measures and gait performance scale (FAC) were significant.

## 4 Discussion

This work describes the changes in functional directed brain connectivity occurring after a rehabilitation treatment in the resting state motor network of a cohort of subjects in subacute stage after unilateral ischemic stroke.

In this work, conversely to other previous EEG-based functional connectivity studies (Pichiorri et al., 2015, 2018), the directed functional connectivity was estimated in the brain source space, since this approach is considered the state of the art in this field, being less prone to volume-conduction effect artifacts and therefore more reliable for connectivity analysis (Michel and Murray, 2012; Haufe et al., 2013; Marinazzo et al., 2019).

The usage of measures derived from the MVAR modeling of time series is frequently employed in the literature to investigate the direction of the information flow in EEG-based connectivity studies with an exploratory approach (Pichiorri et al., 2015, 2018; Lin et al., 2021; Yuan et al., 2021). Usually, one specific measure, e.g., the PDC or its generalized version, is employed to identify the network topology but direct deductions about the transfer of spectral power within connections are not trivial. To overcome this limitation, the main methodological contribution of our study is the combination of complementary information derived from two measures, i.e., DC and gPDC, for a more comprehensive description of the information flow between connections within the motor network. In particular, as the DC represents the fraction of the power in the *i*th series due to the past of the *j*th series, its magnitude provides direct interpretation about the relative importance of the oscillations in the driver signal in causally determining iso-frequency oscillations in the target signal; since the DC accounts for all possible pathways connecting the driver to the target within the analyzed network, it cannot provide information about the activation of specific causal links. On the contrary, the gPDC exploits spectral partialization to elicit causal effects occurring along a specific causal direction, thus highlighting the direct interaction between time series pairs that cannot be attributed to the evolution of other simultaneously observed series. Moreover, comparing DC and gPDC formulations, we observe that the former is a quantity normalized to the information inflow towards the destination *i*th node, whereas the latter is normalized by all the outflows from the *j*th source node. As a consequence, the gPDC magnitude estimation between pairs of nodes (*i,j*) will be reduced if the *j*th source is propagating activity in several directions, with respect to a node propagating its activity only towards one specific direction. Therefore, the gPDC magnitude looses the property of adequately reflecting spectral power, and thus coupling oscillatory content (Faes et al., 2012). As such, the GPDC is mainly employed to identify the strongest significant connections, thus defining the network topography, without a straightforward interpretation on the transferred spectral power. Since in this approach no a-priori hypothesis of a causal structural model is formulated, and given that the network is potentially formed by a full-connected graph, we defined the ICW index to grade the importance of each connection represented for our population of subjects and we identified those connections which were significantly affected by the rehabilitation treatment.

We observed a significant increase of the intra-hemispheric connections’ topographic representation as well as increase in power transfer. In particular, in the α frequency band, we found an enhancement of information flow between pMC and M1 areas in both the ipsi- and contra-lesional hemispheres. On the other hand, in the β frequency band, only the connection from M1 to pMC in the contra-lesional hemisphere showed a significant enhancement in both topographic representation and spectral power. However, we did not find correlations between this intra-hemispheric coupling increase and the motor performance of patients after rehabilitation. In agreement with our findings, the majority of resting-state connectivity studies performed with fMRI in post-stroke populations did not find a strong correlation between changes in intra-hemispheric coupling and motor performance (Grefkes and Fink, 2011; Thiel and Vahdat, 2015). Only one work (Wu et al., 2015) investigating functional connectivity through the coherence of EEG signal found that the increase in ipsilesional connection ipMC–iM1 correlated with increase in FMA scale. It has to be considered that this work investigated the effect of 1-month rehabilitation on patients in chronic stage, hence its findings are not directly comparable with our results on subacute patients. In fact, reorganization of network depends on time after the stroke event. In 2011, Park and others demonstrated time-dependence of increased M1 connectivity with ipsilesional regions and its decreased coupling with contra-lesional ones, showing higher asymmetry at 1 month after stroke (Park et al., 2011).

As for the inter-hemispheric connections, a consistent finding in the literature is that inter-hemispheric coupling between homologous areas is decreased in patients during the acute phase after stroke with respect to healthy subjects. Then, the connectivity progressively increases during the subacute and chronic phases towards pre-stroke coupling levels, documenting a reorganization of resting-state networks (Thiel and Vahdat, 2015). These observations were found using both undirected and directed functional connectivity, exploiting both fMRI (Carter et al., 2009; Van Meer et al., 2010; Wang et al., 2010; Siegel et al., 2016, 2018; Caliandro et al., 2017; Adhikari et al., 2021) and EEG signals (Pichiorri et al., 2015, 2018; De Vico Fallani et al., 2017).

Coherently, based on topographic gPDC analysis, we found an enhancement of the representation of the connection from the ipMC towards the cpMC only in the β frequency range. To be noticed, from our results on DC magnitude, we could not find a significant brain oscillation power transfer between interhemispheric regions of our subacute population. This finding seems an internal discrepancy in our results, but it should be considered that the DC measures take into account not only the power transfer derived from directed connection but also those mediated by other cerebral regions, and this could be a confounding factor.

In the literature, previous studies interestingly found that the variation of inter-hemispheric coupling strength between homologous areas correlates with degree of motor function recovery. This correlation was mainly found between primary motor regions (Grefkes and Fink, 2011; Thiel and Vahdat, 2015), but similar correspondence was observed in an fMRI study at the level of the pre-motor regions (James et al., 2009). Regarding this aspect, we found that the coupling between pre-motor areas from contra-to ipsi-lesional hemisphere assessed in the α band correlates with the improvement of motor performance for the upper limb, even though the increase of cpMC → ipMC connection was not statistically represented in the population. This could be due to the small number of subjects recruited in this study and a lack of homogeneity in the population.

As final interesting result of our analysis, we found a significant increase of topographic representation of connection from SMA toward contra-lesional M1 in α frequency, whereas a decrease of the cpMC → SMA coupling after rehabilitation both in α and β frequency bands was observed. The role of SMA region in stroke recovery is scarcely documented in EEG studies where the functional connectivity is estimated directly from electrodes signals, since SMA and pMC areas are often considered as a single region of interest. In our study, the use of source space localization helped to identify also the SMA as a separate region of interest.

The crucial role of the SMA in motor recovery has been demonstrated in previous task-based fMRI studies on patients with stroke, who exhibit reduced connectivity between the SMA and the ipsilesional pre-motor cortex in both imagined and executed tasks and for which an early involvement of the SMA in the process of stroke recovery and a correlation of the initial activation of the SMA with motor recovery were described (Loubinoux et al., 2003; Sharma et al., 2009). In resting-state directed functional connectivity studies, the coupling between SMA and ipsilesional M1 was demonstrated to be significantly enhanced and correlated with motor function recovery (Rehme et al., 2011; Bajaj et al., 2015). Bajaj and others in 2015 reported also a decrease in the causal flow from right pMC towards SMA, which was modulated by the type of therapy. However, findings are not consistent in literature and this topic needs further investigation.

Our results showed that the information flow through the pre-motor regions can have a relevant role in the reorganization of the motor network during the subacute stage after stroke, involving both intra- and inter-hemispheric couplings. It was hypothesized that with greater motor impairment, secondary motor areas as pMC and SMA are involved in the motor network reorganization to promote motor performance recovery (Ward and Cohen, 2004; Nudo, 2006). The population here investigated showed relevant impairments prior to rehabilitation, probably due also to the early subacute stage in which they were recruited after the stroke event. The severity of paralysis according to the FMA score is distributed as follows: ≤ 25, 26–45, and 46–66 for severe, moderate, and mild paralysis, respectively (Gladstone et al., 2002; Woytowicz et al., 2017). Among the recruited subjects, 9 of them showed serious impairment of the upper limb, seven had a moderate paralysis and only 2 were mildly impaired. In addition, 14 subjects were not independent in walking (FAC_T0_ score equal to 0 or 1). This might explain the great involvement of pMC observed in the recovery phase for these patients, probably more accentuated than in other works were subjects in the chronic phase or with lower impairment were studied.

This work presents also some limitations. The most important one is the small number of patients that we were able to recruit in subacute phase after stroke. Moreover, the high inter-individual variability in lesion location can represent a relevant factor in the significant founding of our population results. In fact, previous studies already reported that the size and location of the lesion, especially if this appears in cortical or sub-cortical brain regions, may influence the reorganization of resting-state networks in stroke recovery (Fanciullacci et al., 2021). Moreover, we want to remark that the signal pre-processing steps and the choice of analysis parameters, e.g., de-noising filters, time-series window length, MVAR model order, electrode or source space, can affect the reproducibility of connectivity results. This aspect is crucial and should deserve further attention towards a standardization.

## 5 Conclusion

In this work, the effects of rehabilitation on directed functional connectivity changes within the motor network’s sensory motor rhythms of sub-acute post-stroke patients was investigated. As compared to previous studies, here a novel methodological approach based on the combination of two measures, i.e., DC and gPDC, was proposed to shed light on both direct and indirect coupling between brain regions and their oscillation frequency properties. The relevant role of the pMC and SMA was particularly highlighted by the topography analysis based on gPDC. In fact, considering only the DC we were not able to identify the changes in SMA connections with contra-lesional motor and pre-motor areas, as well as the enhanced role of the pMC inter-hemispheric coupling in the β band. On the other hand, for intra-hemispheric pMC-M1 coupling, especially in the ipsi-lesional hemisphere, the DC spectral strength transfer seems to be more relevant than its topography representation for the population.

## Data Availability

The datasets presented in this article are not readily available because data is property of Ospedale Valduce, Centro Riabilitativo Villa Beretta. Requests to access the datasets should be directed to the corresponding author.
